# Cavitary Pulmonary Nodules in Rheumatoid Arthritis: A 17-Year Imaging Follow-Up of Necrobiotic Granulomas

**DOI:** 10.7759/cureus.108740

**Published:** 2026-05-12

**Authors:** Abeer Abukamil, Kamil Abushaban, Sayf Al-Katib

**Affiliations:** 1 School of Medicine, Wayne State University School of Medicine, Detroit, USA; 2 Diagnostic Radiology, Corewell Health William Beaumont University Hospital, Royal Oak, USA; 3 Diagnostic Radiology, Oakland University William Beaumont School of Medicine, Rochester, USA; 4 Diagnostic Radiology and Molecular Imaging, Corewell Health William Beaumont University Hospital, Royal Oak, USA

**Keywords:** cavitary pulmonary nodules, computed tomography, granulomatous lung disease, longitudinal imaging, pulmonary necrobiotic granulomas, rheumatoid arthritis, rheumatoid pulmonary nodules

## Abstract

Pulmonary necrobiotic granulomas are a rare extra-articular manifestation of rheumatoid arthritis (RA) that may radiographically mimic infection or malignancy. We present a 57-year-old woman with RA and a smoking history who was found to have subpleural cavitary pulmonary nodules on computed tomography (CT). A biopsy confirmed necrobiotic granulomas.

Serial imaging over a 17-year period demonstrated a fluctuating course, including enlargement of lesions from 1.4 cm to 4.4 cm, development of new cavitary nodules, fluid-filled transformation, and partial spontaneous regression. Despite marked radiographic progression, the patient remained clinically stable and was managed conservatively with multidisciplinary follow-up. To our knowledge, such prolonged longitudinal imaging follow-up of rheumatoid pulmonary nodules is rare.

This case highlights the prolonged and variable natural history of RA-associated pulmonary nodules and underscores the importance of recognizing their imaging characteristics to prevent unnecessary invasive intervention. Long-term surveillance may be appropriate in asymptomatic patients despite radiologic progression.

## Introduction

Pulmonary involvement is a common extra-articular finding of rheumatoid arthritis (RA). The disease can affect all structural components of the lungs, including airways, vasculature, pleura, and most commonly, the parenchyma in the form of interstitial lung disease (ILD) [1-3]. Necrobiotic granulomas, also referred to as rheumatoid pulmonary nodules, are a less common pulmonary manifestation found in 0.2% of chest radiographs of RA patients [4,5]. Histologically, these nodules are defined by central fibrinoid necrosis surrounded by palisading histiocytes and an outer rim of inflammatory and granulomatous cells, including macrophages, lymphocytes, and plasma cells [2,3,6]. Pulmonary necrobiotic granulomas are most commonly found in multiples, usually peripheral and subpleural [7,8]. Prior imaging studies have also demonstrated a strong tendency for nodules to occur in subpleural regions of the lung and to manifest variable morphology, including solid nodules, cavitary lesions, or spontaneous regression over time [7,8]. While they are often asymptomatic, rupture or cavitation of these lesions can lead to complications such as pneumothorax, empyema, pleural effusion, hemoptysis, and bronchopleural fistula [7,9].

Although rare, it is important for radiologists to correctly identify necrobiotic granulomas because they closely mimic infection or malignancy on imaging, including metastatic carcinoma, tuberculosis, and other opportunistic infections [7,8]. Recognition of nodules is important to avoid misdiagnosis and unnecessary invasive procedures. Awareness of this diagnosis in patients with known RA, particularly those with risk factors, such as smoking, allows radiologists to play a key role in guiding multidisciplinary evaluation and management.

## Case presentation

**Figure 1:**
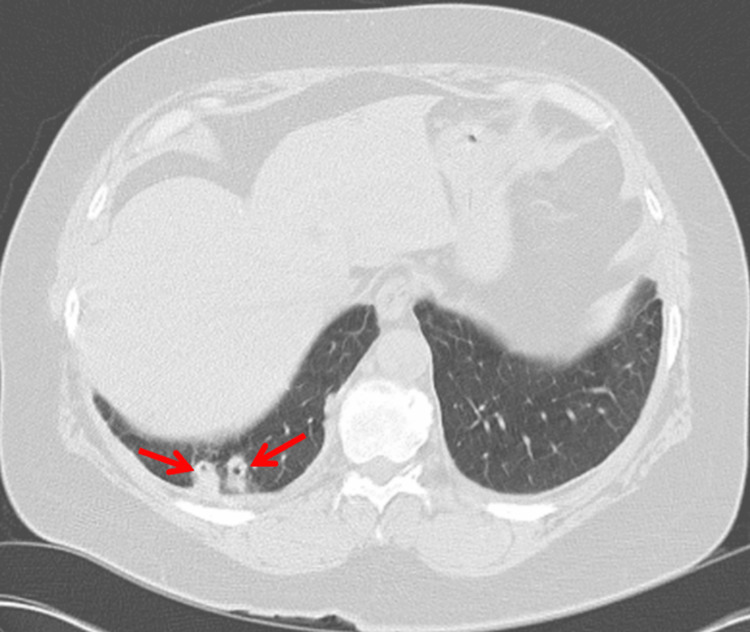
Red arrows showing two thick-walled cavitary subpleural nodules

**Figure 2:**
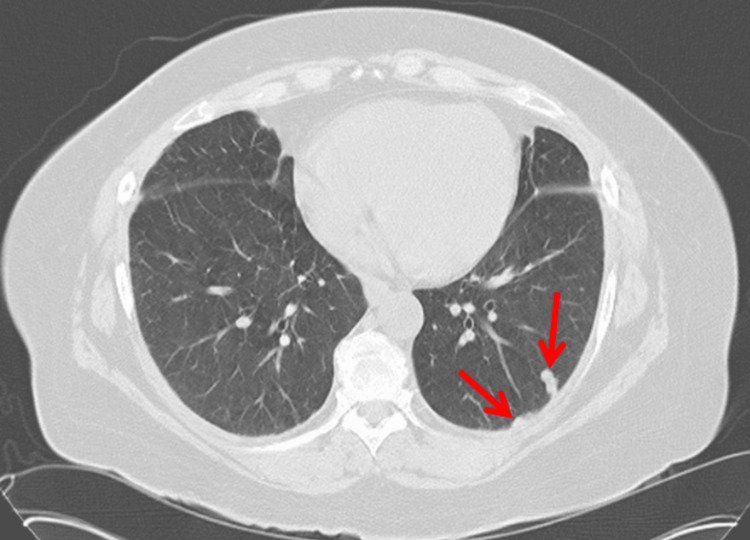
Red arrows showing subpleural nodularity in the left lower lobe

**Figure 3:**
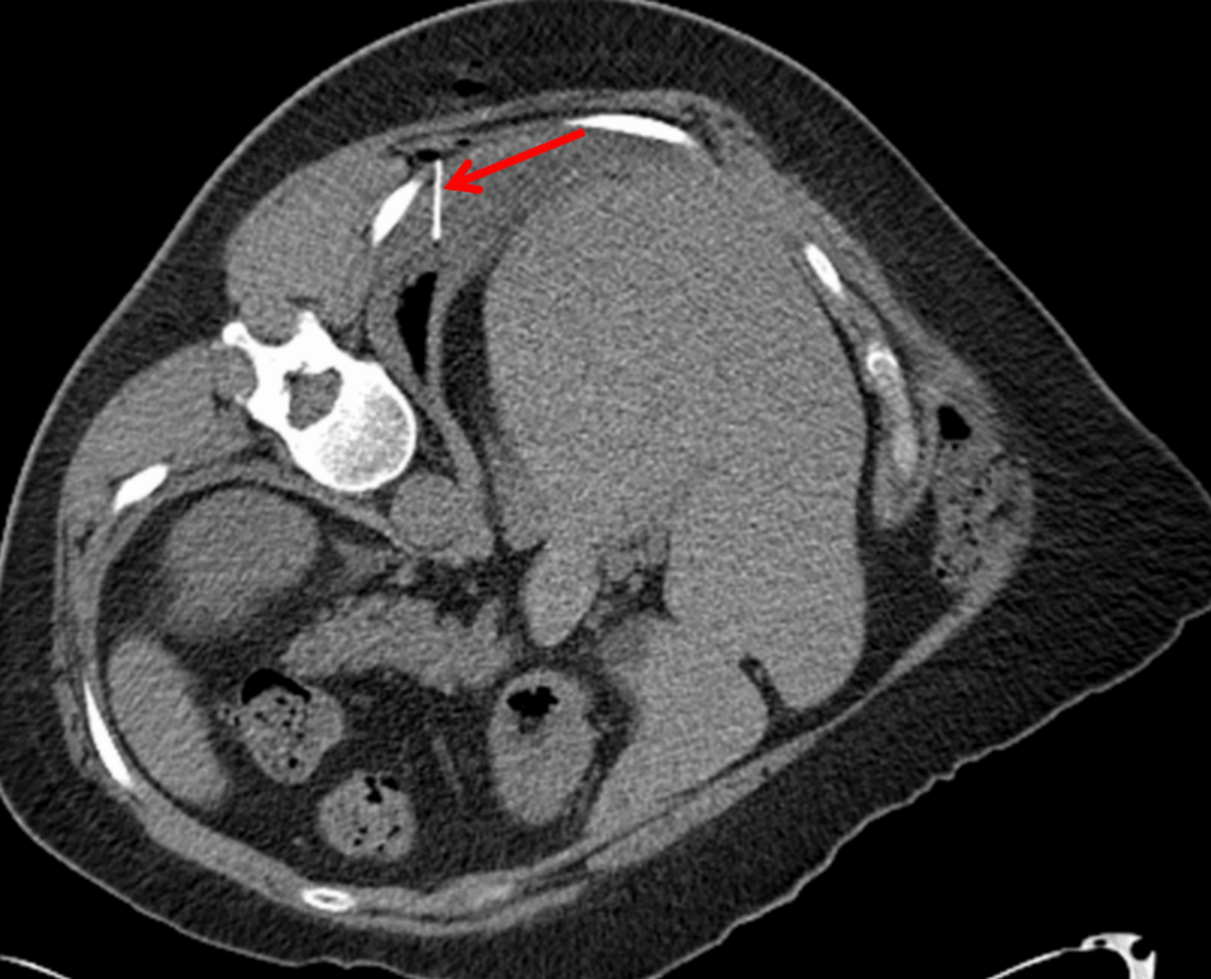
Red arrow showing the biopsy needle in the collapsed cavitary nodule

A 57-year-old female with a past medical history of hypertension, hypothyroidism, rheumatoid arthritis, and smoking presented to the emergency department (ED) for shortness of breath. The patient underwent a CT, which, at the time, demonstrated two thick-walled subpleural cavitary lesions in the right lung base measuring up to 1.4 cm (Figure 1). Additional areas of subpleural nodularity were also present (Figure 2). Given the patient’s smoking status, she underwent biopsy of the right lung base nodules (Figure 3). Tissue pathology came back for necrobiotic granulomas. Lab values were within normal limits, and the remainder of the studies demonstrated no additional abnormalities.

**Figure 4:**
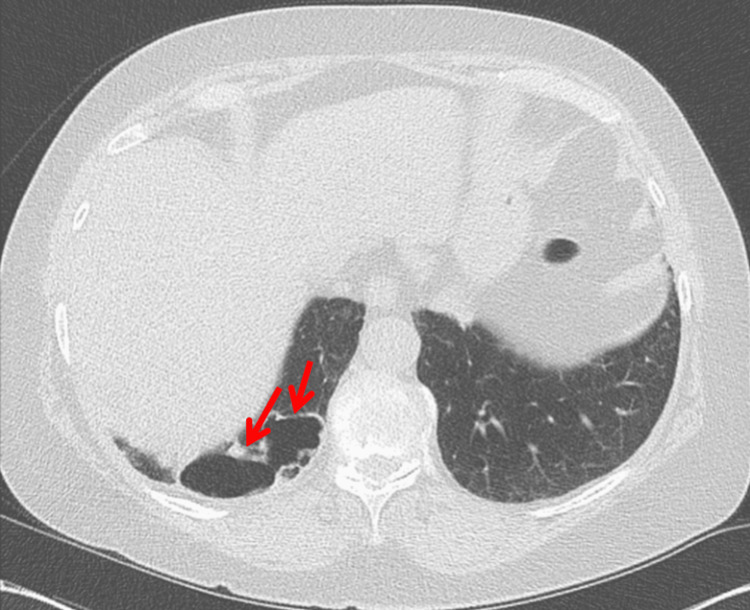
Red arrows showing enlargement of previously biopsied right lung base cavitary lesions

**Figure 5:**
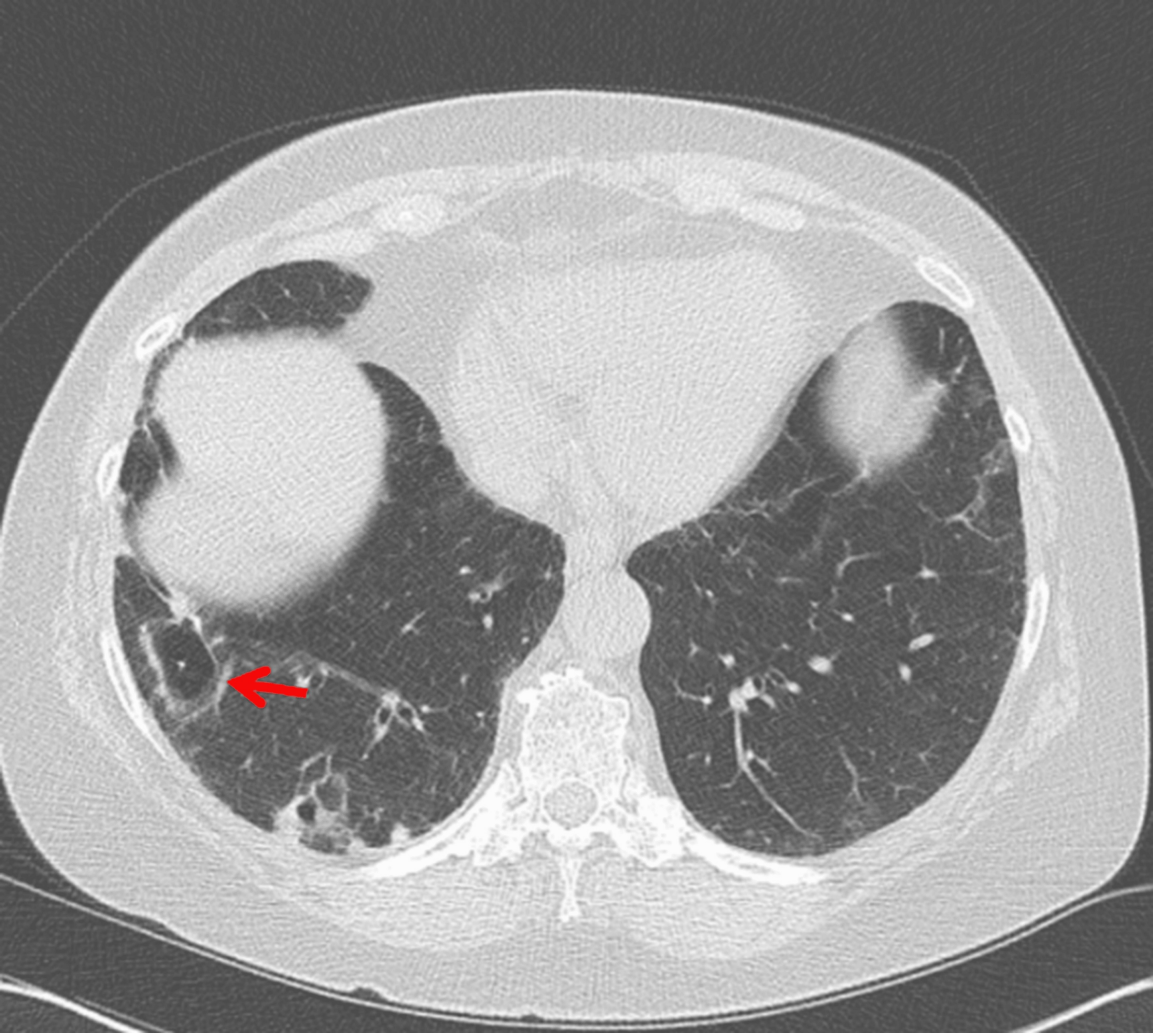
Red arrow showing a new area of cavitation in the right lateral lung base

Eight years following the initial CT and biopsy, repeat low-dose CT for lung cancer screening demonstrated enlargement of previously biopsied subpleural cavitary lesions now measuring up to 4.4 cm (Figure 4) with additional new areas of cavitation (Figure 5).

**Figure 6:**
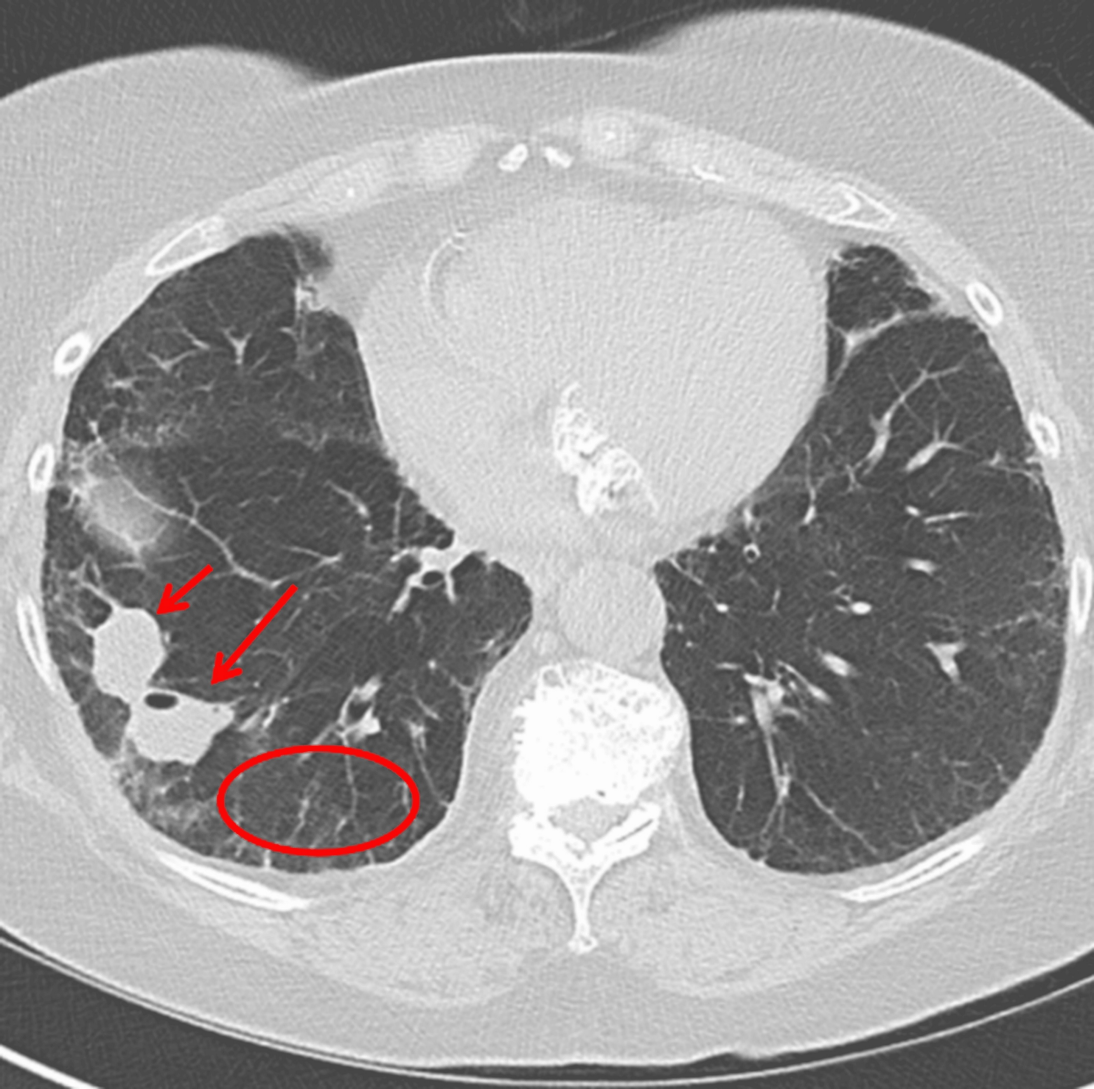
Red circle demonstrating the resolution of the right posterior lung base cavitary lesions; red arrows demonstrating an increase in size of the right lateral lung base cavitary lesion, now filled with fluid

Repeat chest CT obtained for follow-up of these cavitary lesions 17 years post-biopsy demonstrated a decrease in size of posterior right lung base nodules with enlargement of right lateral lung base nodules, now filled with fluid (Figure 6). The patient has been following up with pulmonology and rheumatology for management of her condition.

**Figure 7:**
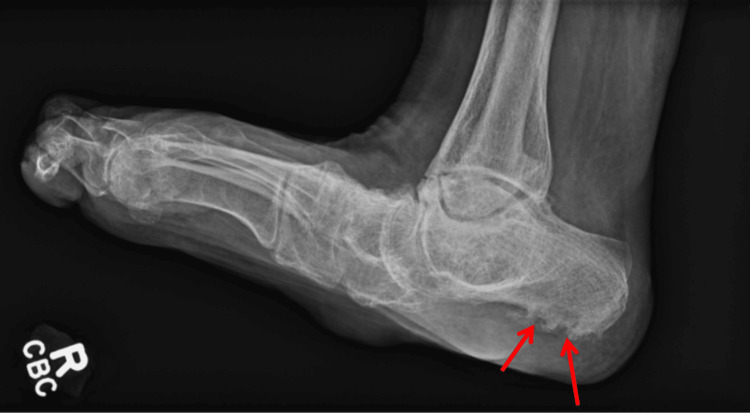
Red arrows demonstrating the erosions seen at the base of the calcaneus, which is characteristic of rheumatoid arthritis

The patient also received foot radiographs for foot pain, which demonstrated erosions in the calcaneus at the site of insertion of the plantar fascia. These erosions are changes consistent with progressive rheumatoid arthritis (Figure 7).

## Discussion

On high-resolution computed tomography (HRCT), pulmonary necrobiotic granulomas characteristically appear as multiple bilateral nodules with a peripheral, subpleural, or periseptal distribution [9]. They most commonly present as solid nodules but frequently undergo central cavitation, producing thick, irregular walls--the pattern demonstrated at initial presentation in this case. Nodule size has a wide range, and while multiplicity is more typical, solitary lesions can sometimes be seen [6,7,9]. Recent reviews of granulomatous lung disease further support that necrobiotic nodules may undergo necrosis, cavitation, and even partial resolution over time, consistent with the longitudinal imaging findings observed in this case [10].

Although prior case series have described similar dynamic behavior, including enlargement, cavitation, and spontaneous regression, as part of an active inflammatory process, the fluid-filled transformation observed at the 17-year follow-up in our patient represents a less commonly documented morphologic change and illustrates the broad CT spectrum these lesions can demonstrate over time [10]. Familiarity with the spectrum of necrobiotic granulomas, from solid to cavitary to fluid-filled, is useful for radiologists when interpreting imaging to avoid mischaracterizing findings as malignant or infectious etiologies.

The primary diagnostic challenge for the radiologist is distinguishing necrobiotic granulomas from other causes of cavitary pulmonary nodules, a differential that includes primary lung malignancy, metastatic disease, septic emboli, and granulomatous infection [11,12]. This diagnostic overlap has been emphasized in several prior studies, noting that rheumatoid nodules may closely mimic metastatic disease or opportunistic infections such as tuberculosis or fungal infection on CT imaging [8,9,13]. Prior imaging studies have also reported that rheumatoid pulmonary nodules most commonly occur in patients with long-standing seropositive rheumatoid arthritis and are frequently associated with cigarette smoking and/or elevated rheumatoid factor titers [5,6,9]. This association is particularly relevant to identify in our patient, as smoking can act as both a risk factor for rheumatoid nodules and a confounder that increases suspicion for malignancy [9,14]. Moreover, broader reviews of thoracic manifestations of RA emphasize that pulmonary nodules represent part of a wider spectrum of RA-related lung disease, such as interstitial lung disease, reinforcing the importance of evaluating these findings within the systemic context of RA at the time of initial diagnostic evaluation [3,14,15].

Comparative imaging studies have identified several features that favor the diagnosis of rheumatoid nodules over malignancy, including multiplicity, smooth borders, cavitation, satellite nodules, and a peripheral or subpleural location, many of which are present in this case [9]. Further indicators that favor necrobiotic granulomas include a waxing and waning course over long periods of time, lack of lymphadenopathy, and, to a lesser extent, bilaterality, subpleural predominance, and lack of pleural invasion. In contrast to infectious cavitary nodules, necrobiotic granulomas typically occur without fever, leukocytosis, or positive cultures, and in the clinical setting of established RA [5,12]. If radiologic findings are indeterminate, radiologic-pathologic correlation is critical. Histopathologic confirmation demonstrating necrobiotic granulomas with central fibrinoid necrosis and palisading histiocytes remains the gold standard for diagnosis of rheumatoid nodules [2,5].

When imaging features are present in a patient with known RA, particularly one with a smoking history, elevated rheumatoid factor, or anti-CCP seropositivity, the radiologist may consider necrobiotic granulomas in the differential and recommend serologic and clinical correlation before proceeding to biopsy [9]. In our case, the initial biopsy was appropriate given the smoking history and the inability to exclude malignancy on imaging alone. This approach is consistent with prior literature stating that while necrobiotic granulomas may be a part of the differential, histopathologic confirmation remains necessary when the clinical context is unclear, particularly in patients with risk factors for malignancy [8,9].

The unique value of this case lies in its 17-year longitudinal CT record. Over this interval, the nodules demonstrated enlargement from 1.4 cm to 4.4 cm, development of new cavitary lesions, and partial spontaneous regression of some lesions with fluid-filled transformation. Previous reports have described similar waxing-and-waning behavior of rheumatoid pulmonary nodules, with lesions occasionally enlarging, cavitating, or regressing over time due to ongoing necrosis as part of the inflammatory process [5,10]. However, most published reports describe shorter imaging follow-up intervals. For example, some case series have reported variable progression over shorter intervals, but few studies have demonstrated continuous imaging evolution spanning more than 10 years, highlighting the rarity and value of prolonged follow-up as demonstrated in this case [10,16]. The tendency for interval enlargement and cavitation to mimic disease progression has been well-documented [9,13]. The changes observed in this case, if interpreted in isolation on any single study, could easily raise concern for malignant progression or superimposed infection. Contextualizing interval change within the established diagnosis of necrobiotic granuloma and recognizing the well-described waxing-and-waning natural history allowed the clinical team to pursue conservative management throughout.

This underscores the radiologist's responsibility not only at the time of initial characterization but across longitudinal follow-up. Recognition of the expected imaging evolution of rheumatoid pulmonary nodules and clear communication of this possibility in radiology reports may help prevent unnecessary invasive procedures or empiric treatments when lesions demonstrate interval change but remain consistent with the known natural history of necrobiotic granulomas. Recent multidisciplinary reviews further stress that collaboration between radiology, pulmonology, and rheumatology is critical in guiding appropriate management strategies and avoiding overtreatment in stable patients.

## Conclusions

This case illustrates the chronic and unpredictable trajectory of RA-associated necrobiotic granulomas. Long-term follow-up in this patient demonstrates the characteristic fluctuating nature of necrobiotic granulomas, including periods of enlargement, cavitation, and spontaneous regression despite minimal clinical symptoms. These findings highlight that radiographic progression does not always correlate with clinical deterioration and support conservative management in asymptomatic patients. Multidisciplinary collaboration between radiology, pulmonology, and rheumatology is essential to guide diagnostic and minimally invasive treatment strategies. Optimal outcomes in RA-associated pulmonary disease focus on patient monitoring, prevention of complications, and preservation of patient functional status rather than aggressive intervention. Recognition of this possible diagnosis and its variable course allows clinicians to avoid unnecessary treatment while prioritizing regular patient follow-ups, reinforcing the importance of longitudinal imaging and patient-centered management in chronic RA-related lung involvement.
